# Deep venous thrombosis in an individual with statin-exposed anti-SRP myopathy: case report and review of literature

**DOI:** 10.1186/s12959-021-00347-x

**Published:** 2021-11-25

**Authors:** Jiali Li, Mingming Yan, Jiao Qin, Lingyan He, Cao Dai, Rui Wen

**Affiliations:** 1 Department of Rheumatology and Immunology, University of South China Affiliated Changsha Central Hospital, 161 South Shaoshan Road, Changsha, 410008 Hunan China; 2 Department of Orthopaedic Surgery, The Second Xiangya Hospital of Central South University, Changsha, Hunan China

**Keywords:** Immune-mediated necrotizing myopathy, Anti-signal recognition particle myopathy, Deep venous thrombosis, Statins

## Abstract

**Background:**

Immune-mediated necrotizing myopathy (IMNM) is characterized by proximal muscle weakness, elvated serum muscle enzyme levels, myopathic electromyography findings, and necrotic muscle fiber with few inflammatory cell infiltration in muscle biopsies. Statins, the first line drug to lower triglyceride and cholesterol level in blood, have been reported to be associated with statins-induced necrotizing autoimmune myopathy (SINAM). Although anti-3-hydroxy-3-methylglutarylcoenzyme-A reductase (anti-HMGCR) myopathy is considered as the leading myopathy related to the statins medication, anti-signal recognition particle (SRP) myopathy were also identified in several cases with statin exposure. The risk of deep venous thrombosis (DVT) is substantially high in individuals with autoimmune inflammatory diseases. But few studies have reported the occurrence and recommendation for treatment of DVT in patients with anti-SRP myopathy. Here, we reported a statin-exposed anti-SRP myopathy individual developed DVT who was successfully treated with catheter-directed thrombolysis (CDT) and systemic anticoagulants therapy.

**Case presentation:**

A 56-year-old Chinese female came to the outpatient room with gradually progressive bilateral lower-extremity weakness. Magnetic resonance imaging revealed myopathy in bilateral thighs. Serum anti-SRP antibody was positive. She was diagnosed with anti-SRP myopathy. When treated with corticosteroids and immunosuppressants, the patient developed mild edema and pain of left lower extremity. Angiography and ultrasound revealed diffuse venous thrombosis of left lower extremity. Therapy was initiated with CDT and lower molecular weight heparin, then switched to once daily oral rivaroxaban. Meanwhile, steroids combined with tacrolimus were also carried on while simvastatin was discontinued. One month later, patient’s symptoms were resolved and only partial thrombosis in left femoral vein was remained.

**Conclusion:**

The prevalence of DVT in patient with anti-SRP myopathy was rare. No well-established treatment strategy is available to manage the IMNM and DVT at the same time. The systemic anticoagulants therapy combined CDT can be an effective therapeutic approach to address extensive DVT in patient with anti-SRP myopathy.

## Background

Immune-mediated necrotizing myopathy (IMNM) is a group of inflammatory myopathies, which is clinically characterized with proximal muscle weakness, elvated serum muscle enzyme levels, myopathic electromyography findings, and necrotic muscle fiber with few inflammatory cell infiltration in muscle biopsies [1]. Multiple causes including autoantibodies, statins administration, paraneoplastic, and viral infection are strongly associated with the IMNM [2]. As the first line drug to lower triglyceride and cholesterol level in blood, statins could cause statins-induced necrotizing autoimmune myopathy (SINAM), which is the mainly side effect responsible for the discontinuation of statins medication [3]. Although anti-3-hydroxy-3-methylglutarylcoenzyme-A reductase (anti-HMGCR) antibody is the most common autoantibodies identified in SINAM, the present of anti-signal recognition particle (SRP) was also confirmed by RNA immunoprecipitation in SINAM [4]. Moreover, it has been showed that anti-SRP antibodies levels correlate with disease activity of SINAM [5]. Therefore, this anti-SRP antibodies can be considered as a specific biomarker to classify the category of SINAM.

As the hallmark feature of SINAM is significant muscle fiber necrosis or regeneration with few lymphocytic infiltration, the prevalence of thrombosis in SINAM is rare compared to other autoimmune diseases such as Churg-Strass syndrome and systemic lupus erythematosus [6, 7]. In addition, the management of DVT in SINAM has not yet been well established. Here, we firstly reported that a patient diagnosed with anti-SRP myopathy developed a severe DVT in left lower extremity. She got a clinical remission after the induction therapy with corticosteroids, immunosuppressants, systemic anticoagulants, and CDT.

## Case presentation

A 56-year-old female with a history of hypertension and hyperlipidemia presented to outpatient room with gradually progressive bilateral lower-extremity weakness more than five weeks and exaggeration for one week. She had difficulty in getting up from the bed and lifting the feet off the floor but denied fever, rash, dysphagia, headache, sialorrhea, diplopia, muscle pain, and sensory changes. There was no family history of genetic myopathies or rheumatologic. She had been taking the amlodipine, metoprolol tartrate, and atorvastatin for 6 years.

A scrotal examination revealed the power in her both upper and lower bilateral proximal extremities was 2/5, and that in both upper and lower bilateral distal extremities was 3/5. Her muscle tone in lower extremities was decreased but deep tendon reflexes were normal.

**Fig. 1:**
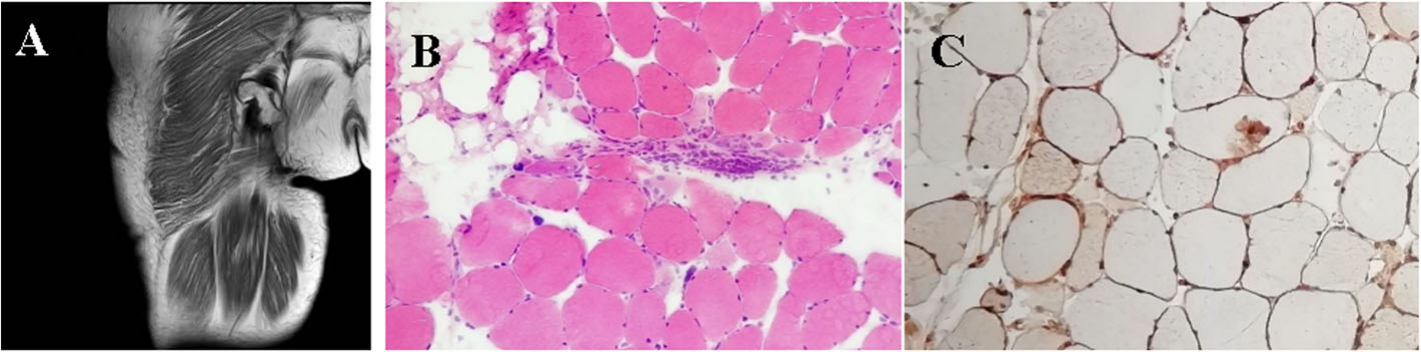
Magnetic resonance image and histological findings of right thigh. **A** Axial T1-weighted femoral MRI of right thigh on admission showed femoral muscle atrophy with fat replacement. **B** Hematoxylin and eosin staining from muscle biopsy sections of right quadriceps showed necrotic muscle occasionally clustered intermingled with small lymphocytes (**C**)

Laboratory tests showed normal complete blood count and C-reactive protein. Sedimentation rate was slightly elevated to 28 mm/h. There was an increase in creatine kinase (CK) 7892 IU/L, myohemoglobin (MYO) 2315 IU/L, and lactic dehydrogenase 1244 IU/L. AST and ALT were increased to 159 IU/L and 171 IU/L, respectively. Serum magnesium were elevated to 1 mg/dL. Her antinuclear antibody and anti-neutrophil cytoplasmic antibody were normal. Serum immunological studies demonstrated postivie antibodies of anti-SRP and Ro-52. Magnetic resonance imaging of thigh revealed extensive edema, suggestive of diffuse myositis (Fig. 1A). Electromyography showed myogenic lesion. A biopsy of muscle of right thigh revealed necrotic muscle clustered intermingled with few lymphocytes (Fig. 1B and C).

She was diagnosed with anti-SRP myopathy. Statin was discontinued. The patient was started on intravenous immune globulin (IVIG) 0.5 g/kg divided over five-day course. Simultaneously, high-dose methylprednisolone (500 mg/d) was administered for three days, followed by solumedorl 80 mg daily. Following the commencement of tacrolimus (3 mg/day), serum CK and MYO levels decreased to 3603 U/L and 2066 U/L, respectively. Muscle weakness gradually recovered. The oral solumedrol upon discharge was slowly tapered down to maintenance dose of 0.8 mg/kg/day.

**Fig. 2:**
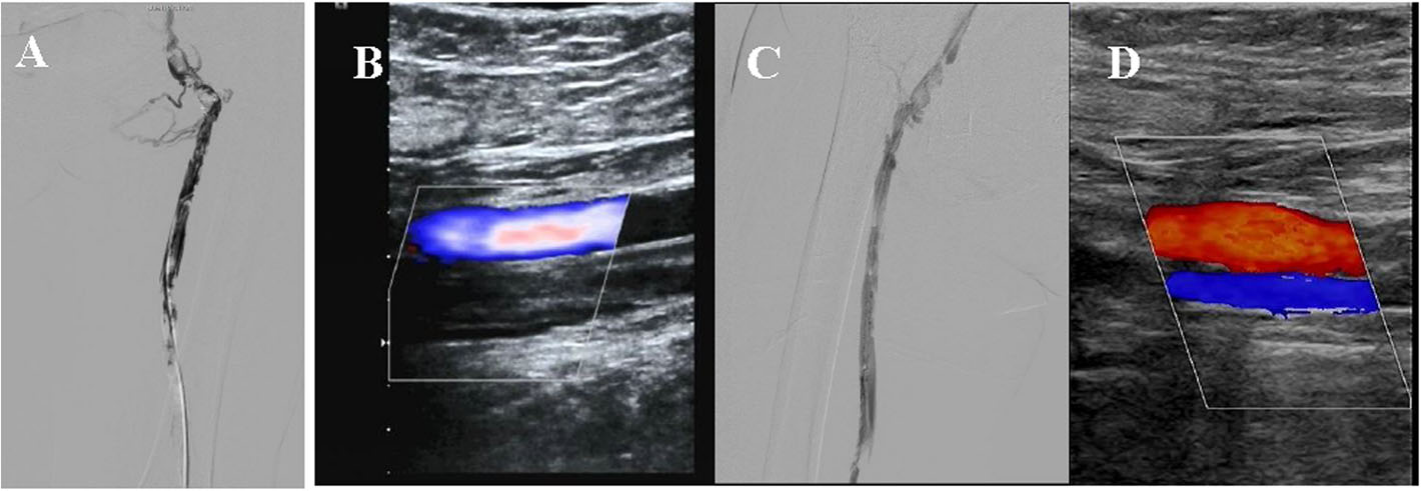
Arterial duplex scan and computed tomography angiography showed the venous thrombosis of left lower extremity before and after anti-thrombosis management. Severe and diffuse venous thrombosis of left lower extremity was confirmed by admission computed tomography angiography ultrasound (**A**) and arterial duplex (**B**). One month after PTA, computed tomography angiography showed a good blood flow with a markedly decrease in length of thrombosis (**C**). Ultrasonography revealed only partial thrombosis in left femoral vein after half year (**D**)

The steroid was slowly tapped down over one month. She was readmitted to our intervention department due to mild edema and pain of left lower extremity. Laboratory tests showed serum CK level decreased to 1241 U/L and serum MYO level decreased to 787 U/L, while D-dimer increased to 22.7 μg/mL. Severe and diffuse venous thrombosis of left lower extremity was confirmed by angiography and ultrasound (Fig. 2A and B). The catheter-directed thrombolysis was performed for revascularization. A self-expandable inferior vena cava filter was deployed and a catheter was percutaneous placed into iliac vein. For thrombolysis, 200,000 U of urokinase (UK) was directly infused twice through catheter within 1 h. After surgery, 400,000 U/day of UK was continuously administrated through catheter for 4 days. Lower weight molecular heparin (5000 U, Q12h) was also injected subcutaneously for five days, followed by orally rivaroxaban 15 mg BID and simvastatin 20 mg qn. To evaluate the efficacy of thrombolysis, percutaneous transluminal angioplasty (PTA) was performed one month after CDT. Angiography revealed partial thrombus removal and good blood flow (Fig. 2C). However, serum CK and MYO levels increased to 4408 U/L and 3681 U/L, respectively. Steroid combined with tacrolimus were carried on while simvastatin was immediately discontinued. With a good response, the power of affected muscle groups was gradually recovered and serum CK level decreased to nearly normal range. After myopathic management, discontinuation of simvastatin, and anticoagulant treatment ultrasonography revealed only partial thrombosis in left femoral vein (Fig. 2D).

## Discussion and conclusions

Autoantibodies recognizing the SRP were first identified in the 1980s, which was reported to be highly associated with disease activity of IMNM [8]. Patients with positive anti-SRP myopathy have more severe proximal muscle weakness, higher number of necrotic muscle fibers, more common interstitial lung disease compared to anti-HMGCR myopathy [9]. Statins are associated with a number of myalgia and myopathy including IMNM. Statin-associated myopathy is mainly related with anti-HMGCR antibodies [10]. However, about 20% anti-SRP myopathy patients have been reported to have statin exposure [11]. After 5 years of atorvastatin medication, our patient developed anti-SRP myopathy with severe proximal muscle damage. With a good response to the immunosuppressive treatment, she had a clinical remission. However, serum CK levels sharply rose when simvastatin was prescribed to treat DVT, which further supported that the occurrence of anti-SRP myopathy was not limited to specific category of statins administration.

This is the first case to report DVT in patients with anti-SRP myopathy. The risk of DVT is substantially high in patients with autoimmune inflammatory diseases. According to Virchow’s triad, three main plausible existing mechanisms including venous stasis, increased coagulability of blood and vessel wall damage contribute to the high risk of DVT [12], which is also applied to patients with anti-SRP myopathy. First of all, the patients with anti-SRP myopathy suffer from serious muscle weakness which may lead to venous stasis due to decreased mobility [13]. Moreover, inflammation is able to modulate thrombotic responses by upregulating procoagulants and damaging vessel wall. However, as anti-SRP myopathy is characterized by myofiber necrosis over inflammation [1], venous stasis should be the primary risk factor of DVT. In addition, the use of steroid, which helps to slow the progression of the disease, could be the another driver of the increased risk of DVT [14].

Given the presumptive mechanisms discussed above, the primary recommendation for DVT should be myopathic treatment. At present, there are no clinical trials to guide therapeutic decision in anti-SRP myopathy. The recommendation derived from most recent European Neuromuscular Center criteria for IMNM suggests that corticosteroids plus immunosuppressant is considered to be an initial treatment [15]. High-dose corticosteroids or IVIG could achieve an excellent response in the most severely affected individuals. In this case, a combination of immunosuppressive therapy (eg., high-dose solumedorl, tacrolimus, and IVIG) achieved a satisfactory clinical outcome. However, she had an acute proximal DVT of the leg on the second admission, which was likely due to simvastatin-induced anti-SRP myopathy. The recommendation for DVT management revealed that anticoagulant therapy plus CDT to restore venous patency is much more effective than anticoagulation alone [15] . Due to the efficacy of selective thrombolysis and reduced hemorrhagic complication compared with systemic infusion, CDT has been an appearing management for DVT [16]. Therefore, we applied CDT combined with both heparin infusion and oral rivaroxaban to counter DVT, which contributed to a full thrombosis remission. In the absence of other studies on management of anti-SRP myopathy and DVT at the same time, this report provide a substantial value for physicians to improve clinical outcome of similar cases.

## Data Availability

All data generated or analyzed during this study are included in this published article.

## Abbreviations

IMNM: Immune-mediated necrotizing myopathy
SINAM: statin-induced necrotizing autoimmune myopathy
HMGCR: 3-hydroxy-3-methylglutarylcoenzyme-A reductase
SRP: signal recognition particle
DVT: deep venous thrombosis
CDT: catheter-directed thrombolysis
CK: creatine kinase
MYO: myohemoglobin
IVIG: intravenous immune globulin
PTA: percutaneous transluminal angioplasty
